# Scholarly activity as a selection criterion in the Canadian Residency Matching Service (CaRMS): A review of published criteria by internal medicine, family medicine, and pediatrics programs

**DOI:** 10.36834/cmej.69094

**Published:** 2020-07-15

**Authors:** Jorin Lukings, Amanda Bell, Karl Stobbe, Vesa Basha, Jessie Brazier, Delia Dragomir, Meghan Glibbery, Hannah Kearney, Alison Knapp, Daniel Levin, Dyon Tucker, Seddiq Weera, Larry W. Chambers

**Affiliations:** 1 Niagara Regional Campus, Michael G. DeGroote School of Medicine, Faculty of Health Sciences, McMaster University, Ontario, Canada

## Abstract

**Background:**

Undergraduate medical students seek as much information as possible as to how residency programs select candidates. The Canadian Residency Matching Service (CaRMS) website is one of their primary sources of information. Students may be more competitive in the match if they know whether scholarly activity is used in the selection process by their preferred programs, as described on the CaRMS website.

**Methods:**

For all 17 Canadian faculties of medicine, 2019 R1 entry internal medicine, family medicine and pediatrics program descriptions were reviewed on the CaRMS website looking for keywords related to scholarly activity.

**Results:**

Forty-one percent of family medicine, 65% of internal medicine and 71% of pediatric programs explicitly stated having interest in applicants with scholarly experience. In Western Canada, 80% of internal medicine and 60% of pediatrics programs included scholarly activity in their CaRMS description of criteria considered in ranking applications. Similarly, in Ontario, 66% of internal medicine and 83% of pediatrics programs mentioned scholarly activity as a valuable quality. In Quebec 100% of family medicine and 50% of pediatrics programs include scholarly activity in their descriptions. Pediatrics and family medicine programs (100%) in Atlantic Canada mentioned scholarly activities but neither of the two Atlantic Canada internal medicine programs mentioned scholarly activities.

**Conclusion:**

Undergraduate medical students can use this project to prioritize extracurricular activities and scholarly work to be competitive for application to family medicine, internal medicine and pediatrics residency programs.

## Abstract (fr)

**Contexte:**

Les étudiants de premier cycle en médecine cherchent autant de renseignements que possible sur la manière dont les programmes de résidence sélectionnent les candidats. Le site Web du Service canadien de jumelage des résidents (CaRMS) représente l’une de leur principale source de renseignements. Les étudiants peuvent être plus compétitifs dans le jumelage s’ils savent comment les activités d’érudition sont utilisées dans le processus de sélection par leurs programmes préférés, comme il est décrit sur le site Web de CaRMS.

**Méthodes:**

Pour l’ensemble des 17 facultés de médecine canadiennes, les descriptions des programmes d’entrée R1 de 2019 en médecine interne, en médecine familiale et en pédiatrie ont été examinées sur le site Web du CaRMS pour la recherche de mots clés liés aux activités d’érudition.

**Résultats:**

41 % des programmes de médecine familiale, 65 % de ceux en médecine interne et 71 % de ceux en pédiatrie énonçaient explicitement avoir un intérêt pour les candidats avec une expérience en érudition. Dans l’Ouest canadien, 80 % des programmes en médecine interne et 60 % de ceux en pédiatrie comportaient une activité d’érudition dans leur description CaRMS des critères dont ils tenaient compte dans le classement des demandes. De même, en Ontario, 66 % des programmes en médecine interne et 83 % de ceux en pédiatrie mentionnaient que les activités d’érudition représentaient une qualité importante. Au Québec, 100 % des programmes en médecine familiale et 50 % de ceux en pédiatrie présentent une activité d’érudition dans leur description. Les programmes de pédiatrie et de médecine familiale (100 %) dans le Canada atlantique mentionnent les activités scientifiques, mais aucun des deux programmes de médecine interne du Canada atlantique ne mentionnait des activités scientifiques.

**Conclusion:**

Les étudiants de premier cycle en médecine peuvent utiliser le présent projet pour prioriser leurs activités extracurriculaires et le travail d’érudition pour être compétitifs lors de leurs applications dans des programmes de médecine familiale, de médecine interne et de pédiatrie.

## Introduction

Understanding how the Canadian Residency Matching Service (CaRMS) matching process operates is of great interest to Canadian undergraduate medical students. The Canadian residency match has significant implications for medical students in terms of their future geographical location, personal lives, and specialty in which they will practice.^1^^-^^5^

A 2015 review of 20 published studies found scholarly activity such as research and quality improvement of health services experience increases the likelihood of a student matching into their residency of choice, regardless of what that may be.^6^ This is accomplished by developing useful clinical and interpersonal skills while closely interacting with the speciality of choice^6^. Students who participate are more likely to attend conferences, which serve as opportunities for networking and can produce connections that open doors on the residency program interview trail. Additionally, scholarly activity pairs the student with a dedicated mentor. This connection itself can be instrumental in a student’s professional growth and development during medical school.

CaRMS is the corporation that runs the residency match. However, the application evaluation process that determines which candidates will be admitted to residency programs is carried out by each school’s medical program. CaRMS does not make decisions about program requirements or candidate selection criteria. CaRMS only receives the ranking data and runs the algorithm which ranks the applicants. Matches are made based on congruence of the ranking of candidates by the residency programs and the ranking of residency programs by candidates.

As provincially funded residency positions become more competitive,^7^ it is important that candidates know which activities and attributes each residency program values most in their candidate. We do not know how many programs include participation in scholarly activities in their description to potential candidates as a desirable attribute.

Canadian Medical Education Directives for Specialists (*C*anMEDS)^8^^,^^9^ considers scholarly activity to be an essential competency of practicing physicians. According to CanMEDS, scholarly activity should be a mandatory component of physician education in addition to the other seven core competencies: medical expert, communicator, collaborator, manager, health, advocate and professional. According to CanMEDS, scholarly activity involves being:

- Engaged in the continuous enhancement of their professional activities through ongoing learning
- Teaching students, residents, the public, and other health care professionals
- Integrating best available evidence into practice
- Contributing to the creation and dissemination of knowledge and practices applicable to health.^10^

The selection criteria used by residency programs across Canada are multi-faceted and incorporate information from written applications, reference letters and interviews with applicants as they rank candidates for their program. However, the extent to which ‘scholarly activity’ is considered in the selection process is unclear. Applicants to residency programs wish to know the criteria that give them the best chance of being highly ranked by the residency programs of their choice. CaRMS applicants may better understand the selection process if residency programs explicitly identify ‘scholarly activity’ as an asset in their published 2019 CaRMS program descriptions. Students may be more competitive in the residency match if they know whether scholarly activity is used in the selection process by their preferred programs, as described on the CaRMS website.

## Methods

Data for this project were collected with the assistance of eight medical students (VB, JB, DD, MG, HK, AK, DL, DT) reviewing published residency program descriptions for R1 entry for the 2019 match year on the CaRMS website. All 51 residency program descriptions were reviewed for internal medicine, family medicine and pediatrics in Canada’s 17 medical schools looking for specific keyword descriptors related to research and scholarly activity. The internal medicine and family medicine programs have the largest number of residency spots and pediatrics was chosen as the third program as an example of a smaller program. A review of the ethics of this study was not required as it was based on publicly available data from the CaRMS website.

Residency program descriptions that specifically mentioned the keyword ‘scholarly activities’ or similar descriptors such as ‘applied health research’, ‘clinical research’, ‘quality improvement’ or ‘education research’ in their program descriptions were identified.

Both French and English terms were captured. For those terms that had the same meaning in French and English, for example,’ research’ and ‘recherche’, only the English versions were reported. If French terms did not show up in the list of English terms, they were left in the list of all descriptors.

The medical students were given an orientation about the goals and objectives of the project. They were instructed not to include the provincial criteria for selecting residents and criteria from the Royal College of Physicians and Surgeons of Canada and the Canadian College of Family Physicians.

The students were also instructed on how to complete the Data Retrieval Form. The Form is a table with the cells used to record the descriptors.

The students retrieved explicit descriptors of ‘scholarly activity’ reported in the CaRMS website for the residency programs assigned to them. The students worked in four pairs with each member of the pair first independently assessing four to five residency program CaRMS descriptions. The pairs then came together to discuss any discrepancies by comparing their list for each program description that they reviewed. These discussions between the pairs of students were used to resolve inconsistencies in their two lists of descriptors for each program they reviewed.

The data were then analyzed to compile a list of all descriptors used in reference to scholarly activity and the frequency with which each descriptor appears in each residency program description on the CaRMS website. The data were summarized using a tag cloud (‘WordCloud’) which provides a visual representation of the language used to describe any requirements related to scholarly activity in the residency program descriptions. The tags are single words or phrases and the frequency of each tag (word or phrase) used by residency programs is shown with font size and colour.

The frequency of scholarly activity-related language in each of the residency program CaRMS website descriptions was summarized by program and by region of Canada. This avoided having to reveal results from individual programs. We hypothesized that there would be a similar amount of variation in scholarly activity requirements across programs as in regions of Canada.

## Results

**Figure 1:**
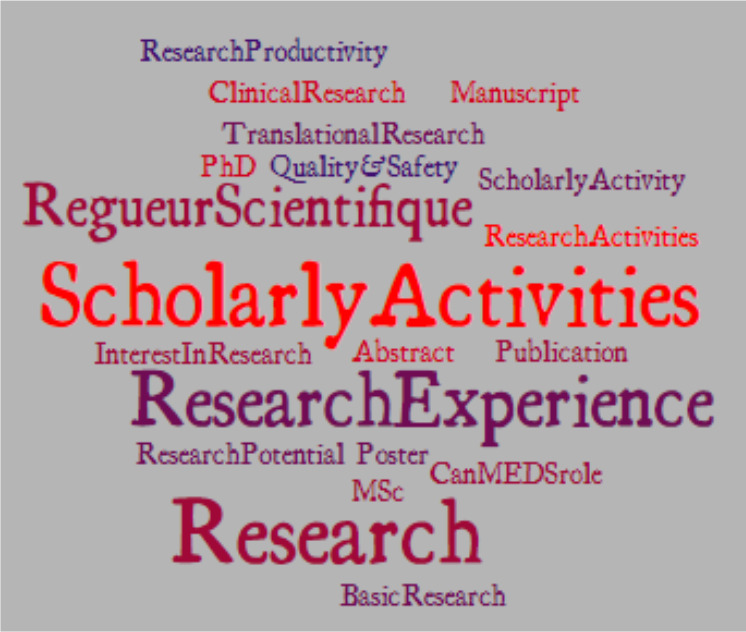
Word Cloud descriptors of scholarly activity as a selection criterion in the 2019 Canadian Residency Matching Service (CaRMS) website for the internal medicine, family medicine, and pediatrics residency programs

The word cloud (please see Figure 1) revealed the terminology commonly used by residency programs when describing scholarly activity on the CaRMS website. Residency programs across the three specialities – family medicine, internal medicine and pediatrics – used variable language to describe applicants’ scholarly accomplishments. ‘Research’ was the most common and ‘scholarly activity’ the second most common phrases or words. Some mentioned the completion of an MSc and a PhD are valuable assets. Although ‘Quality Improvement’ was included in the 2015 review as an activity that improved the likelihood of successful residency match,^6^ ‘Quality and Safety’ did appear but ‘Quality Improvement’ as a scholarly activity did not appear in any of the program descriptions.

**Figure 2:**
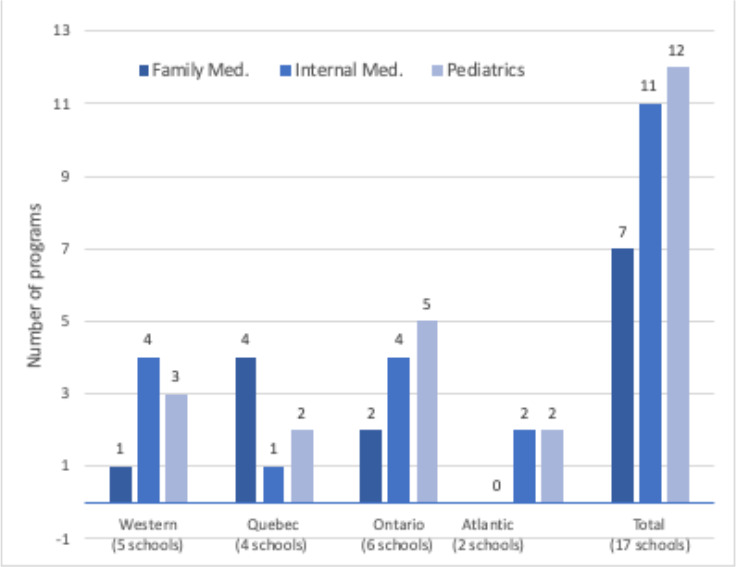
Number of 51 family medicine, internal medicine and pediatrics residency programs mentioning scholarly activities in the 2019 CaRMS website by region of Canada.

The review of 51 Canadian R1 residency programs across 17 medical schools revealed that 41% (7 of 17) of the family medicine, 65% (11 of 17) of the internal medicine and 71% (12 of 17) of the pediatric residency programs explicitly stated their interest in applicants with scholarly activities (see Figure 2).

In five medical schools in Western Canada, as shown in Figure 2, 80% (4 of 5) of the internal medicine programs, 60% (3 of 5) of the pediatrics programs and 20% (1of 5) of the family medicine programs included scholarly activity in their CaRMS description of criteria considered in ranking applications. Similarly, for the six medical schools in Ontario, 67% (4 of 6) of the internal medicine, 83% (5 of 6) of the pediatrics residency programs and 33% (2 of 6) of the family medicine programs mentioned scholarly activity as a desirable accomplishment. In four medical schools in Quebec 100% of the family medicine (4 of 4), 50% (2 of 4) of the pediatrics programs and 25% (1 of 4) the internal medicine programs have scholarly activity in their descriptions. While the family medicine programs in the two medical schools in Atlantic Canada did not mention scholarly activities, the pediatrics and internal medicine programs both (100%) did mention scholarly activities.

## Discussion

This review of R1 residency program descriptions demonstrated that the majority of internal medicine and pediatrics programs and minority of family medicine across the country include scholarly activity in their descriptions of criteria considered in ranking candidate applications. This trend is reversed, however, in Quebec programs where all the family medicine, half of the pediatrics programs and one quarter of the internal medicine programs include scholarly activity as an attribute. The range of words used to describe scholarly activity was found to be broad and non-specific which makes it difficult for candidates to know which types of activities will be most highly valued and how they should present their interests and work to best meet the program expectations.

The description of scholarly activities for select residency programs varied from program to program and generally lacked specificity. Some programs suggested an interest in research would qualify as scholarly activity. The completion of an MSc or PhD during medical school can be quite difficult or not possible unless the student is given special leave of absence or acceptance into limited MD/PhD program spots. Other programs mention graduate degrees as important demonstration of scholarly work. Indeed, some schools, such as the University of Toronto, encourage undergraduate MD students to enrol in a MSc or PhD program while studying medicine. Other undergraduate MD students may have an MSc or PhD before they begin studying medicine. Some residency programs are intentionally structured to allow for completion of graduate degrees during the residency and this may be a more productive or relevant time to pursue these studies.

From this review of the 2019 CaRMS website, it appears that students would more easily be able to interpret what qualifies as scholarly activity if residency programs adhered to a uniform manner of describing scholarly activity in their CaRMS website outlines. This would be reassuring to some applicants who do not have this interest. However, the CanMEDS classification of scholarly activity as a core competency of practising physicians would demonstrate that training and skill in this area should be accomplished by all medical students. There also is a risk that a student will be disadvantaged in their residency applications by not having scholarly activity as part of their curriculum vitae when a program may be valuing these skills, despite not explicitly mentioning them in their online description. Without describing the range of scholarly activity that would be considered, including quality improvement activities or work in curriculum development, students may not consider the work they have done to be “research” and may have concerns they do not meet the program’s threshold for scholarly activity.

Thus, based on the review of the 2019 CaRMS website, it would benefit both applicants and residency programs to introduce standard program descriptions that include uniform descriptions of selection criteria for residency programs concerning all the essential physician competency requirements as outlined in *C*anMEDS, including scholarly activities.

This project supports the changes made in the 2020 CaRMS website. Residency programs are now required to report in the descriptions of their program on the CaRMS website whether “research/publications” are considered in their review of documents and whether ‘scholarship’ will be a topic covered in interviews of residents. While this has forced programs to be more explicit about these topics being considered in the admission process, the ways in which residency programs use this information can be the subject of further research.

One limitation of this project is that it included only reviews of family medicine, internal medicine and pediatrics residency programs. Internal medicine and family medicine programs have the largest number of available positions in Canada. Pediatrics, a smaller residency program had a different value placed on scholarly activity in their selection criteria and this may be true with other smaller residency programs. Future reviews could examine other programs on the CaRMS website. There is a possibility that programs did not include a discussion of desired scholarly activity on their CaRMS online description despite valuing this in candidate ranking. This disadvantages students in their application. There also may be more detailed program descriptions available on the university website of each program and future studies could look at the concordance between the CaRMS program description and the university website description for individual residency programs.

## Conclusion

The results of this project support the revisions to the 2020 CaRMS website that now requires programs to comment on whether ‘research/publications’ are considered in reviewing applicant documents and whether ‘scholarship’ will be discussed in the applicant’s interview. Our project can assist students in organizing and prioritizing their MD training activities in a manner that aids them during residency applications. Also, the project results were forwarded to CaRMS and the Canadian Federation of Medical Students.

Authorship: JL, AB, KS, SW and LWC contributed to the conception or design of this project VB, JB, DD, MG, HK, AK, DL and DT obtained the data for this project. Analysis and interpretation of the data involved all the authors. All the authors were involved in drafting the work or revising it critically for important intellectual content; All the authors approved the version of the manuscript to be published. All the authors take responsibility for being accountable for all aspects of the work in ensuring that questions related to the accuracy or integrity of any part of the work are appropriately investigated and resolved.
